# Supplementary material for: The Transcription Factors AcuK and AcuM Influence Siderophore Biosynthesis of Aspergillus fumigatus
Source: J Fungi (Basel). 2024 Apr 30;10(5):327. doi: 10.3390/jof10050327 (PMC11121910; doi:10.3390/jof10050327)
Supplement: Supplementary file 1 [file jof-10-00327-s001.zip › jof-2942589-supplementary.pdf]

SUPLEMENTARY MATERIAL

Table S1: Strains used in this study.

Strain	Description	Reference
A1160+	CEA10; *ΔakuB*^{ku80}*::pyrG⁻zeo, pyrG⁻::pyrG*^{Af}*; MAT1-1*	[39]
ΔacuM	A1160+; *acuM::hph*	[38]
ΔacuK	A1160+; *acuK::hph*	[38]
ΔacuE	A1160+; *acuE::hph*	This study
AfS77	ATCC4664; *ΔakuA::loxP*	[41]
ΔsidA	AfS77, *ΔsidA::six*	[7]

Table S2: Primers used in this study.

Primer	5′-3′ Sequence
oPC48	TAAATTGCATTTTTGATCTTGAAT
oPC49	TAGTTCTGTTACCGAGCCGGgttgatattgtcttttgcaa
Hph-fw	ccggctcggtaacagaactaACGGCGTAACCAAAAGTCAC
Hph-rv	gggagcatatcgttcagagcTCTTGACGACCGTTGATCTG
oPC50	GCTCTGAACGATATGCTCCCcctgcaagattgtgagcgtg
oPC51	ACTGGGATGTGGTGCGGGGCGGAG
oPC52	CATCGGAACAAGTCGGACAAG
oPC53	GAGATATTCAAGAAGTTGTAGAGGATG

Table S3: Primers used for the amplification of the digoxigenin-labelled probes for Northern- and Southern blot analysis.

Probe	Gene	5′-3′ Sequence
hapX	AFUA_5G03920	TCG GTG GAA AGA AGT GCC<br>CGA CGA TGT ATT GTT ATT GG
mirB	AFUA_3G03640	AAGCCGAGAAAAAGGGGG<br>AACCCAGATGAAGCCCAG
mirD	AFUA_3G03440	TGATGCAGGGGAAATGCTGC<br>TGATATAGGGAGAGGAGGTAAAGGCG
sidA	AFUA_2G07680	AACTACCTCCACCAGAAG<br>GAACGGCAATGTTGTAAG
sit1	AFUA_7G06060	AGAACCAACCATGAACATGGCGATGCAC<br>TTACGGATGATTTATCATCAATCTCCTCCG
sidF	AFUA_3G03400	CCT CAT CCC TAT CTC ACC<br>AGT TTT GAG CGA GAG GGG
ftrA	AFUA_5G03800	ATGGCAAAAGACGTATTTGC<br>TCAGACAAGGGATGCTC
facA	AFUA_4G11080	TTCGTCGAAGGTCGCTTGAA<br>CTCATCGACTCAGGAGCGAC
acoA	AFUA_6G12930	CAG CGT CCT CTC ACA TAC<br>GCA AGA ACC GAT CAG ACC
acuE	AFUA_6G03540	CAATGCTCTGAATGCGGACG<br>GGTGCTTGCGGAACTTCTTG
acuN	AFUA_6G06770	TTGGCCCTGCTCTCATCAAG<br>TGACGTCGGCCTTGTAGAAC
acuD	AFUA_4G13510	TCTGAAGCCGCAACACTCAT<br>TCCAGTTGAAGGATGGCGAC
acuF	AFUA_6G07720	GTCTTCGACCCTATCAGCCG<br>TTTAGCAGCTCAGAGGGCAC
acuG	AFUA_4G11310	ACATTGCTCTGCCATGCTCT<br>TTCATGTTGGGGTGGGTCAG
3′NCR *acuE*	AFUA_6G03540	GCTCTGAACGATATGCTCCC<br>ACTGGGATGTGGTGCGGGGCGGAG
5′NCR *fcyB*	AFUB_025700	CAGAGAATTGCCAAGCTGGT<br>GTGGTGGGGATTGACTCAG
3′ NCR *fcyB*	AFUB_025700	TGCGGTTTTTGGGTTTTATC<br>AGACCGTTGTTTCATACCGC

Table S4: Absolute values of experiments presented in Figure 2. The TAFC content of the supernatant was normalized to g biomass, the ferricrocin content of the mycelia was normalized to 80 mg.

	Strain	Biomass (g) -Fe	Biomass (g) +Fe	TAFC (Abs/g)	Ferricrocin (Abs/80mg)
**Gln**	wt	0.079	0.335	0.570	0.103
		0.080	0.289	0.525	0.100
		0.077	0.336	0.558	0.099
	ΔacuM	0.056	0.202	0.268	0.073
		0.064	0.174	0.234	0.071
		0.061	0.147	0.262	0.075
	ΔacuK	0.071	0.25	0.324	0.064
		0.068	0.182	0.353	0.075
		0.070	0.183	0.386	0.072
**NH_4^+**	wt	0.077	0.155	0.545	0.110
		0.078	0.167	0.462	0.096
		0.086	0.158	0.523	0.117
	ΔacuM	0.064	0.118	0.266	0.080
		0.069	0.139	0.232	0.076
		0.064	0.132	0.234	0.079
	ΔacuK	0.064	0.121	0.278	0.071
		0.063	0.138	0.222	0.080
		0.067	0.136	0.228	0.073

Table S5: Absolute values of experiments presented in Figure 5. The TAFC content of the supernatant was normalized to g biomass, the ferricrocin content of the mycelia was normalized to 80 mg.

		Glucose				Fructose			
	**Strain**	**Biomass (g) -Fe**	**Biomass (g) +Fe**	**TAFC (Abs/g)**	**Ferricrocin (Abs/80mg)**	**Biomass (g) -Fe**	**Biomass (g) +Fe**	**TAFC (Abs/g)**	**Ferricrocin (Abs/80mg)**
**-Acetate**	wt	0.077	0.155	0.545	0.110	0.067	0.14	0.343	0.039
		0.078	0.167	0.462	0.096	0.066	0.142	0.333	0.041
		0.086	0.158	0.523	0.117	0.069	0.143	0.333	0.040
	ΔacuM	0.064	0.118	0.266	0.080	0.055	0.11	0.236	0.037
		0.069	0.139	0.232	0.076	0.057	0.114	0.228	0.033
		0.064	0.132	0.234	0.079	0.056	0.113	0.214	0.032
	ΔacuK	0.064	0.121	0.278	0.071	0.058	0.113	0.207	0.033
		0.063	0.138	0.222	0.080	0.058	0.112	0.224	0.034
		0.067	0.136	0.228	0.073	0.057	0.116	0.211	0.032
**+Acetate**	wt	0.075	0.162	0.627	0.269	0.068	0.141	0.397	0.064
		0.080	0.166	0.413	0.272	0.069	0.14	0.391	0.063
		0.083	0.16	0.470	0.267	0.068	0.144	0.412	0.060
	ΔacuM	0.069	0.134	0.275	0.231	0.058	0.134	0.293	0.068
		0.068	0.143	0.338	0.221	0.057	0.121	0.263	0.064
		0.072	0.14	0.292	0.223	0.059	0.13	0.271	0.066
	ΔacuK	0.063	0.142	0.317	0.209	0.057	0.126	0.281	0.063
		0.067	0.139	0.343	0.207	0.058	0.127	0.276	0.060
		0.069	0.146	0.290	0.207	0.062	0.128	0.290	0.066

Table S6: Absolute values of experiments presented in Figure 8. The TAFC content of the supernatant was normalized to g biomass, the ferricrocin content of the mycelia was normalized to 80 mg.

	Strain	Biomass (g) –Fe	Biomass (g) +Fe	TAFC (Abs/g)	Ferricrocin (Abs/80mg)
**Glucose**	wt	0.072	0.189	0.444	0.099
		0.069	0.192	0.435	0.090
		0.069	0.193	0.435	0.097
	ΔacuE	0.073	0.196	0.438	0.099
		0.071	0.192	0.465	0.098
		0.072	0.195	0.417	0.094
**Fructose**	wt	0.072	0.152	0.389	0.065
		0.064	0.13	0.391	0.060
		0.069	0.145	0.406	0.061
	ΔacuE	0.069	0.141	0.377	0.061
		0.069	0.126	0.406	0.059
		0.068	0.135	0.397	0.064

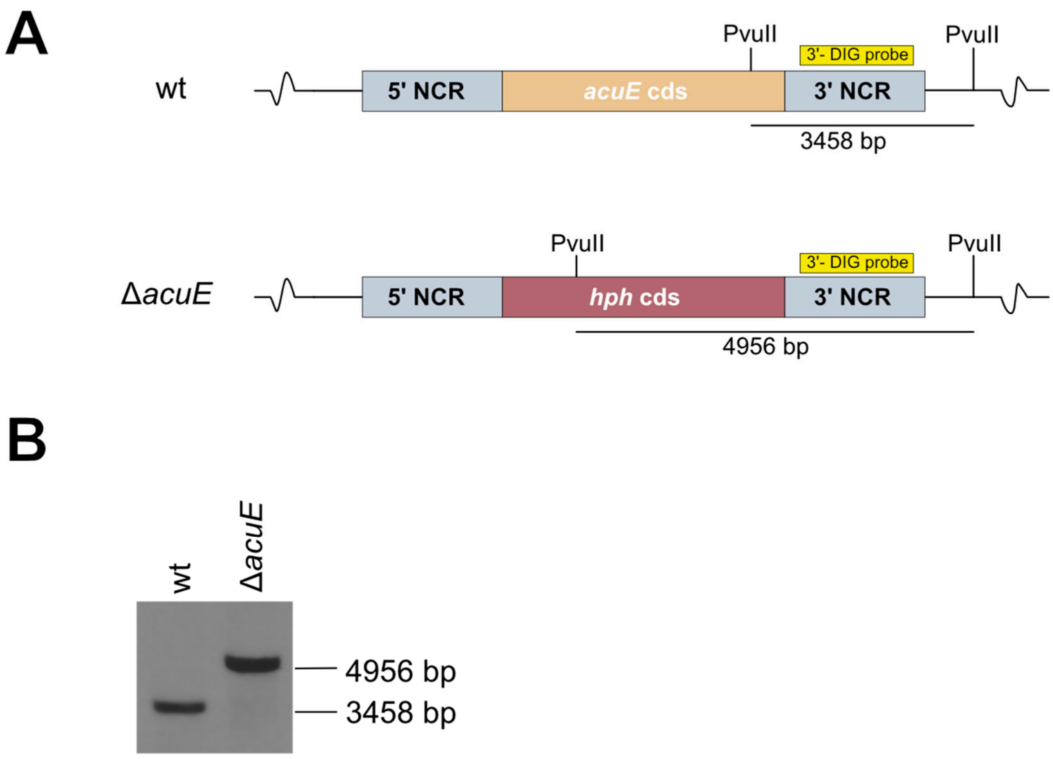

Figure S1: Scheme for deletion of *acuE* in *A. fumigatus* (A) and Southern blot analysis verification (B). Digestion with the restriction enzyme *Pvu*II results in 3458-bp fragment in wt strain and a 4956-bp fragment in *ΔacuE*. The hybridization probe labelled in A was generated from gDNA using primers PC50/PC51. NCR, non-coding region.

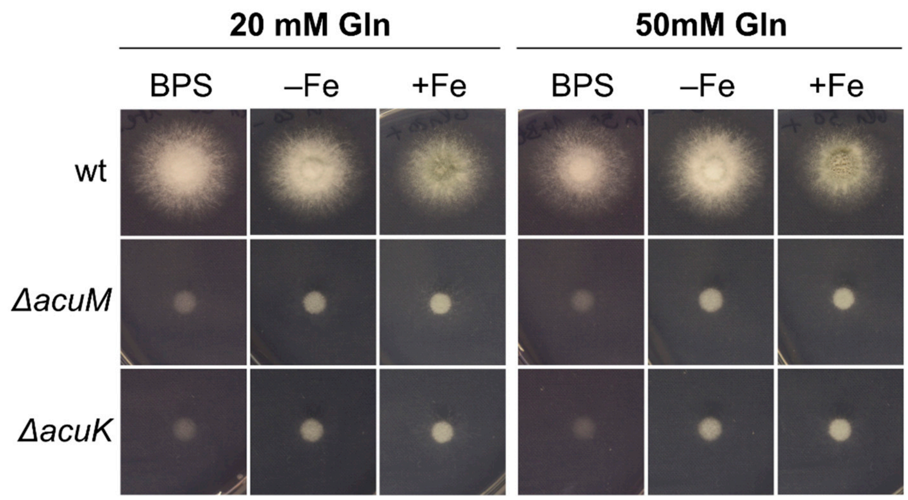

Figure S2: Mutants lacking either AcuM or AcuK are able to slightly use Gln as a carbon source. *Aspergillus fumigatus* wt and *ΔacuM* and *ΔacuK* mutant strains were point-inoculated using 10^4 spores on minimal medium using either Glc or acetate as carbon sources and Gln or NH_4^+ as nitrogen sources. Experimental details are described in Figure 1.

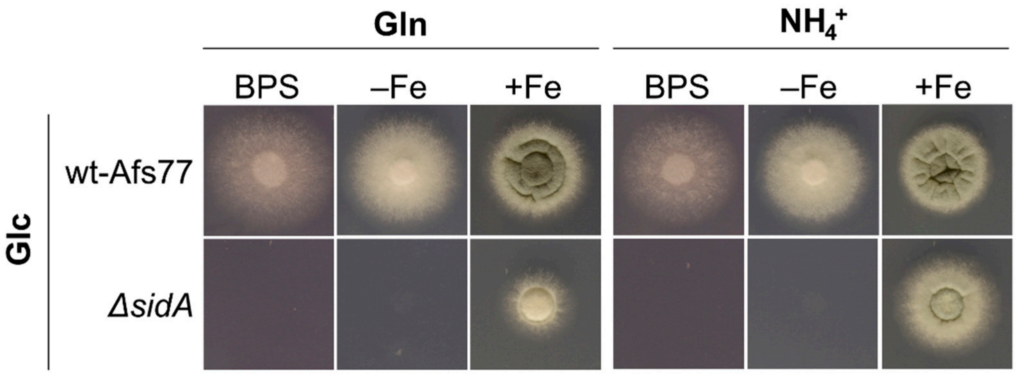

Figure S3: Siderophore-lacking *ΔsidA* mutant displays a growth defect under both iron starvation (-Fe) and in the presence of BPS. *Aspergillus fumigatus* wt and *ΔsidA* mutant strains were point-inoculated using 10^4 spores on minimal medium using either Glc or acetate as carbon sources and Gln or NH_4^+ as nitrogen sources. Experimental details are described in Figure 1.

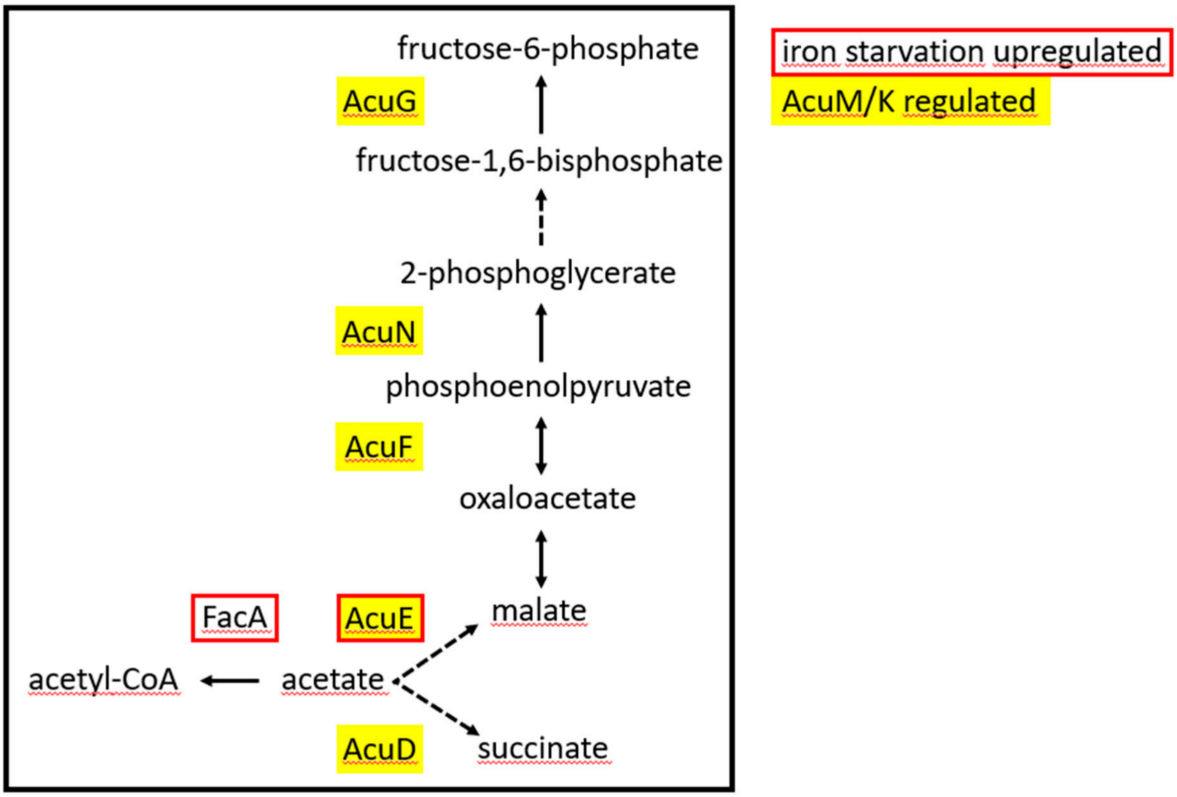

Figure S4: Metabolic interaction of the enzymes encoded by the genes analyzed in Figure 6. Upregulation of the encoding gene during iron limitation compared to iron sufficiency is marked by boxing in red; activation by AcuM and AcuK is marked in yellow
